# Laughter and Death (Poetry)

**Published:** 1882-03

**Authors:** 


### LAUGHTER AND DEATH.

There is no laughter in the natural world
Of beast or fish or bird, though no sad doubt
Of their futurity to them unfurled
Has dared to check the mirth-compelling shout.
The lion roars his solemn thunder out
To sleeping woods. The eagle screams her cry;
Even the lark must strain a serious throat
To hurl his blest defiance at the sky.
Fear, anger, jealousy have found a voice.
Love's pain or rapture the brute bosoms swell.
Nature has symbols for her nobler joys,
Her nobler sorrows. Who had dared foretell
That only man, by some sad mockery,
Should learn to laugh who learns that he must die?

